# Facility or Transport Inequality? Decomposing Healthcare Accessibility Inequality in Shenzhen, China

**DOI:** 10.3390/ijerph19116897

**Published:** 2022-06-04

**Authors:** Zhuolin Tao, Qi Wang

**Affiliations:** 1 Faculty of Geographical Science, Beijing Normal University, No. 19, Xinjiekouwai Ave., Haidian, Beijing 100875, China; taozhuolin@bnu.edu.cn; 2 Proficiency Skill Appraisal and Guidance Center of Natural Resources Ministry, Beijing 100830, China

**Keywords:** healthcare accessibility, inequality, facility distribution, transport, travel time

## Abstract

Accessibility to healthcare services is crucial for residents’ wellbeing. Numerous studies have revealed significant spatial inequality in healthcare accessibility across various contexts. However, it still remains unclear whether the inequality is caused by the unbalanced spatial distribution of healthcare facilities or by unequal transport access to them. This study decomposes inequality in healthcare accessibility into facility- and transport-driven inequality by comparing scenarios of healthcare accessibility, which consider various combinations of multidimensional components of accessibility using different distance measures. Using a case study in Shenzhen, this study reveals that both facility distribution and transport access substantially contribute to spatial inequality in healthcare accessibility. Facility distribution accounts for 61.3% and 50.8% of the overall accessibility inequality for driving and transit modes, respectively. The remaining inequality is induced by imbalanced mobility provided by transport networks. Furthermore, the impact of transport component on healthcare accessibility is unevenly distributed. This study highlights that both facility- and transport-related countermeasures should be considered to improve the accessibility and equality of healthcare services. It provides transferable methods for quantitatively decomposing facility- and transport-driven inequality in accessibility to healthcare or other facilities.

## 1. Introduction

Accessibility to public services (e.g., healthcare, education, and public parks) is crucial for residents’ quality of life and wellbeing and has drawn considerable attention from both policy makers and researchers. Accessibility is a multidimensional concept that can be interpreted and measured from various perspectives [1]. For example, accessibility can be classified into potential versus revealed accessibility, or spatial versus non-spatial accessibility [2]: potential accessibility refers to the potential opportunities for residents to reach services, whereas revealed accessibility is about the actual utilization of services; spatial accessibility is focused on the spatial barriers and available means of transport, whereas non-spatial accessibility mainly pay attention to socioeconomic factors. Accessibility can be also classified into place-based or individual-based accessibility [3]. The former refers to the accessibility to services from a given location, whereas the latter is focused on the influences of individuals’ attributes on accessibility. In this study, accessibility is mainly interpreted as potential spatial accessibility and place-based accessibility. Numerous studies have revealed obvious spatial disparities and inequality in accessibility to healthcare and other facilities across various contexts [4,5,6], from developed to developing countries [6,7,8] and across various geographic scales [9,10,11].

Promoting equality in accessibility to healthcare services is a worldwide policy goal. In China, the equalization of essential healthcare services is considered one of the most important national strategic goals [12]. The operational definition and quantitative measurement of equality in healthcare services have emerged as key to these goals. Healthcare accessibility is generally defined as the opportunities for residents at various locations to obtain healthcare services provided by facilities [13,14,15]. An increasing number of studies propose to measure the spatial equality of public services based on the disparity in accessibility across space or among socioeconomic subpopulations [16,17,18,19]. Equally distributed accessibility is considered a competent representation of the “equality of opportunities” [20,21,22]. In recent years, the COVID-19 pandemic has drawn great attention, not only to the allocation of and access to healthcare services [5], but also to the travel behaviors of residents [23,24]. Notably, such travel behavior impacts of the pandemic are inequal across subgroups with various socioeconomic conditions [25]. These changes highlight the urgent need to investigate the interplay between transport mobility and healthcare accessibility.

Accessibility to facilities depends on various factors, such as the distribution of facilities, the spatial configuration of transport networks, and the complex interactions between them [13,14,26]. As pointed out by Geurs and van Wee [1], accessibility has several components, among which the land use component and the transportation component are closely related to spatial accessibility. In the field of healthcare services, the land use component refers to the distribution of facilities and its relationship with population distribution, whereas the transportation component refers to the transport networks connecting facilities and population. 

Many methods have been developed for measuring spatial accessibility [13,14,16]. Among them, the gravity-based models, including the gravity model and the two-step floating catchment area (2SFCA) model, can comprehensively integrate the facility distribution and transport components [27,28]. In these measures, the influence of transport component on healthcare accessibility is reflected by the variable of travel cost. Although Euclidean distance and road network distance have been adopted in earlier studies, travel time is widely considered as a better representation of travel cost in measuring accessibility [29]. Recent studies further emphasize that the disparity in travel times between different transport modes has significant impacts on healthcare accessibility and equality [30,31,32]. Some studies made efforts to compare the equality of healthcare accessibility under various transport modes [11,33]. Improved versions of 2SFCA have also been developed to incorporate multiple transport modes into one model by considering the competition among various modes [34,35,36]. Some other studies investigated the influences of changes in transit networks on healthcare accessibility and equality [37,38]. These studies suggest that multiple transport modes have an important role in healthcare accessibility and equality. However, since accessibility is shaped by various components simultaneously, it is challenging to separate the contributions of these components [39]. A recent study made progress in isolating the contribution of transport component to accessibility disparity by comparing the accessibility based on travel time to that based on Euclidean distance [39]. However, the study was focused on individual-based accessibility, rather than place-based accessibility. The latter has a closer connection with transport and facility configuration components, and is more widely applied in urban planning and policy making. There is still a research gap in quantifying the contributions of the facility distribution and transport components to the spatial inequality in healthcare accessibility.

Given the above research gaps, this study aims to propose a method for decomposing the contributions of facility distribution and transport networks (both road and transit networks) to the spatial inequality in healthcare accessibility. To achieve this goal, various scenarios of healthcare accessibility using different distance measures are set up and calculated. These scenarios reflect different combinations of the factors in healthcare accessibility. The spatial inequality of healthcare accessibility can be decomposed by comparing these scenarios. The methods will be illustrated and validated using a case study in Shenzhen, China.

The remainder of this paper is organized as follows. Following the introduction, Section 2 develops the methodology and introduces the study area and data for the case study. Section 3 presents the results. The discussion and conclusions are given in Section 4 and Section 5, respectively.

## 2. Materials and Methods

### 2.1. Scenarios for Decomposing Spatial Inequality in Accessibility

**Figure 1:**
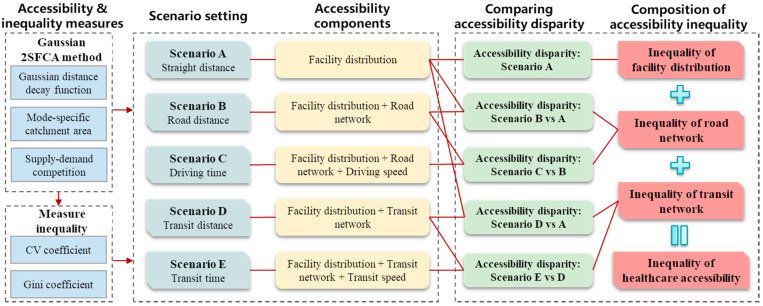
Research framework in this study.

In this study, the spatial inequality in accessibility is understood and measured in terms of the disparity in accessibility across locations. The spatial inequality in healthcare accessibility might be caused mainly by two factors. The first factor is the distribution of facilities, which often mismatches with the distribution of the population to a certain extent. The second factor is the spatial configuration of multimodal transport networks, which would further reshape the spatial relationship between demanders and facilities. Furthermore, transport-related factors can be classified into transport network-related and travel time-related components. Enlightened by the approach to isolate the contribution of transport factor to individual-based accessibility developed by [39], this study proposes that the contributions of these factors to accessibility inequality can be separated by comparing various scenarios with different distance measures. Each scenario is related to a unique combination of accessibility components. Specifically, as shown in Figure 1, the following five scenarios are set up for analysis in this study.

Scenario A uses straight (Euclidean) distance as the measure of distance in calculating accessibility. By doing so, only the first component of accessibility disparity, i.e., facility distribution, is included in the analysis. This component of accessibility inequality can thus be separated from other components.

Scenarios B and C both measure healthcare accessibility by driving mode. Scenario B measures accessibility based on road network distance, which considers not only the impact of facility distribution, but also that of the spatial configuration of the road network. Scenario C uses real-world travel time by driving as the measure of distance, which further incorporates the influence of variable travel speeds on different roads. By comparing Scenarios B with A and C with B, these two components of driving mode accessibility inequality can be separated.

Scenarios D and E both measure accessibility by public transit. Similarly, Scenario D adopts transit network distance, whereas Scenario E uses door-to-door travel time by transit. By comparing Scenarios D with A and E with D, these two components of transit-mode accessibility inequality can be separated.

### 2.2. Measuring Healthcare Accessibility

As demonstrated earlier, healthcare accessibility is influenced by various components, such as the spatial separation between population and facilities and the transport means for conquering such separation [13]. These components collectively determine accessibility to healthcare facilities and its spatial disparity. Therefore, to achieve the goal of this study, the measure of healthcare accessibility should consider these essential components. Among the widely used measures of accessibility, the two-step floating catchment area (2SFCA) method has been renowned for its comprehensive consideration of supply-side factors, demand-side factors, and the transport connections between the two sides [14,16]. The original 2SFCA has been criticized due to overlooking accessibility variation within the catchment area [40]. Some improved forms of 2SFCA introduce an additional distance decay function [14,19,41].

In this study, the Gaussian 2SFCA, which incorporates a Gaussian-form distance decay function [19,42], is applied to measure healthcare accessibility in each scenario. The advantages of Gaussian 2SFCA are three-fold. First, it adopts a moderate distance decay trend. Second, only one parameter, i.e., the size of the catchment area, is required to apply the model. Third, it inherits the property that the population-weighted average accessibility is identically equal to the ratio of total supply to total population [27,28]. This property assures that the accessibility calculated in various scenarios are comparable.

According to Gaussian 2SFCA, accessibility can be calculated in two steps. In the first step, the resources provided at each facility are allocated to all demand nodes within its catchment area. The allocation is based on the demand size at each node and its distance from the facility. It generates the supply-to-demand ratio for each facility:

(1) $$R_{j}=\frac{S_{j}}{\sum_{k\in \left\{d_{kj}\leq \;D_{0}\right\}}P_{k}f\left(d_{kj},\;D_{0}\right)}$$

where $R_{j}$ represents the supply-to-demand ratio at facility *j*, $S_{j}$ is the size of supply of facility *j*, $P_{k}$ is the size of demand at location *k*, $d_{kj}$ is the distance from *k* to *j*, and$\;D_{0}$ is the radius of catchment areas.

In the second step, the supply-to-demand ratios for facilities within the catchment area of each demand node are summed, from which the accessibility at demand node *i* ($A_{i}$) is calculated:

(2) $$A_{i}=\underset{j\in \left\{d_{ij}\leq D_{0}\right\}}{\sum }R_{j}f\left(d_{ij},D_{0}\right)$$

The distance decay function $f\left(d_{ij},D_{0}\right)$ takes a Gaussian form, which has only 1 parameter that needs to be set [19,43]. It can be written as:

(3) $$f\left(d_{ij},D_{0}\right)=\{\begin{array}{c} \frac{e^{-1/2\times {\left(d_{ij}/D_{0}\right)}^{2}}-e^{-1/2}}{1-e^{-1/2}},\;\;\;d_{ij}\leq D_{0}\\ \;0,\;\;\;\;\;\;\;d_{ij}>D_{0} \end{array}$$

In this study, the distance between demand and facility (*d_ij_*) is measured in various forms, including Euclidean distance, transport network distance, and travel time. Euclidean distance is estimated using the Point Distance tool in ArcGIS 10.7. Travel distance and time by driving or public transit are estimated using the navigation Application Programming Interface (API) of Baidu Map. The Driving Navigation API provides reliable estimation of travel time by driving, which considers real-world driving rules and real-time traffic congestion. The Transit Navigation API estimates door-to-door travel time by public transit, which considers all available transit modes in the given city (e.g., metro, bus), the service frequency and transfers, and the walking distance to and from the stations. Therefore, the online map API is widely considered a promising approach to estimating travel time [42,43]. In addition, the above two APIs generate both travel time and travel distance for the optimal routes.

For a given origin–destination pair, the travel costs measured in different distance forms differ from each other. This difference makes it difficult to set up a one-size-fits-all parameter of catchment area size. Therefore, this study sets the catchment area size as the average value of the travel cost for all community-facility pairs. This criterion is unified across scenarios, but can yield heterogeneous catchment area sizes for different types of distances. By doing so, the catchment areas of facilities would be relatively comparable in terms of geometry areas across various scenarios. This setting can generate comparable accessibility and inequality results across scenarios. The catchment area sizes for the five scenarios (A to E) are 22 km, 32 km, 44 min, 34 km, and 95 min, respectively.

### 2.3. Metrics for Measuring the Spatial Inequality of Accessibility

Many indices can be used for measuring spatial inequality, among which the coefficient of variation (CV) and Gini coefficient (GC) are widely applied. The former is defined as the ratio of the standard deviation to the mean value, whereas the latter measures the extent to which the distribution of an indicator deviates from the equal distribution. A larger value for two metrics indicates a higher inequality. CV can be calculated by:

(4) $$CV=\frac{\sqrt{\sum_{i}{\left(A_{i}-\bar{A}\right)}^{2}}}{\bar{A}}$$

where $A_{i}$ is the accessibility score of unit *i*, and $\bar{A}$ is the mean value of the accessibility scores.

**Figure 2:**
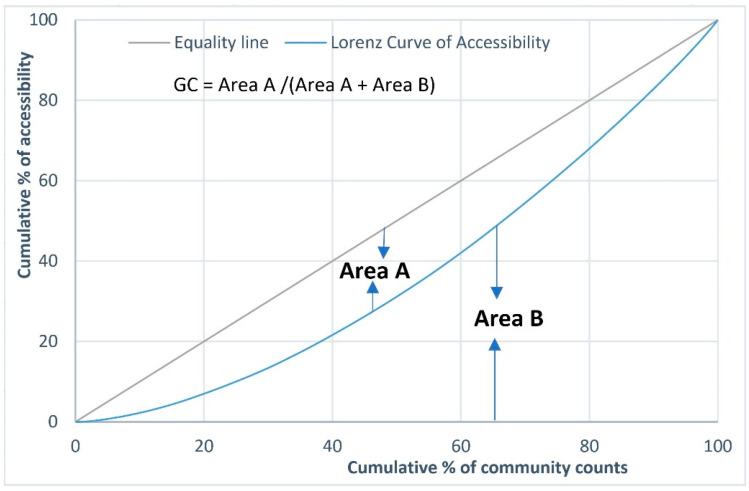
Illustration of Lorenz curve and Gini coefficient.

GC can be calculated by:

(5) $$GC=\frac{\sum_{i,j}\left|A_{i}-A_{j}\right|}{2n^{2}\bar{A}}$$

where $A_{i}$ and $A_{j}$ are the accessibility scores of units *i* and *j*, respectively, $\bar{A}$ is the mean value of the accessibility scores across all units, and *n* is the number of units. Figure 2 shows an example of the Lorenz curve and the corresponding GC, where the Lorenz curve represents the cumulative distribution of accessibility across all units. Area A is the area between the Lorenz curve and the equality line, whereas Area B is the area below the Lorenz curve. In this example, GC can be calculated by dividing A by the sum of A and B.

The GC (or CV) of accessibility by travel time (driving or transit mode) represents the total accessibility inequality for each mode. By comparing GCs in different scenarios for each mode, healthcare accessibility inequality can be decomposed into two parts: facility- and transport-driven accessibility inequality. The latter can be further decomposed into transport network- and travel time-driven accessibility inequality. The procedures are conducted for driving and transit modes, respectively. Taking driving mode as an example, GCs in straight-distance, road-distance, and driving-time scenarios (i.e., scenarios A, B, C) are denoted as ${\mathrm{GC}}_{A}$, ${\mathrm{GC}}_{B}$, and ${\mathrm{GC}}_{C}$. Then, the proportions of facility distribution-, transport network-, and travel time-driven accessibility inequality for the driving mode can be calculated as ${\mathrm{GC}}_{A}$/${\mathrm{GC}}_{C}$, (${\mathrm{GC}}_{B}-{\mathrm{GC}}_{A}$) /${\mathrm{GC}}_{C}$, and (${\mathrm{GC}}_{C}-{\mathrm{GC}}_{B}$) /${\mathrm{GC}}_{C}$, respectively.

### 2.4. Study Area and Data

**Figure 3:**
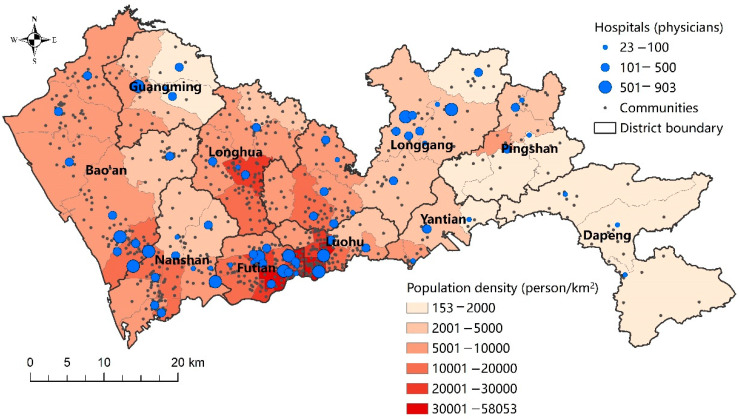
The distribution of hospitals and administrative divisions in Shenzhen.

Shenzhen is a prefecture-level city in Guangdong Province, China, with a population of 17.56 million permanent residents. Our analyses are conducted based on the basic spatial units in Chinese cities, i.e., communities. There were 771 communities in Shenzhen in 2016 under the jurisdiction of 67 subdistricts and 10 districts. The community-level population data were collected from the sixth national population census data in 2010, while the subdistrict-level population data in 2016 were collected from the statistical yearbook of each district. Since more updated community-level population data are unavailable, the community-level population in 2016 were estimated based on the population growth trends at the subdistrict level from 2010 to 2016. In other words, in the estimation, uniform population growth rate was assumed for communities within each subdistrict. The location of each community is set as the location of the administration office of the community, due to the lack of population data at finer scale. In general, the Futian, Luohu, and Nanshan districts are considered the central city of Shenzhen. Figure 3 shows the distribution of subdistrict-level population density in Shenzhen. It is densest in Luohu and Futian, followed by Nanshan, Longhua, Bao’an, and western Longgang.

For healthcare facilities, public general hospitals are included for analysis in this study for two reasons. First, public hospitals play a dominant role in the provision of healthcare services in China [44]. Second, general hospitals provide services to a relatively large proportion of the population within a certain distance. Therefore, both driving and public transit are taken for visits to general hospitals. The list, addresses, number of physicians and beds, and other attributes of general hospitals were obtained from the Shenzhen Municipal Health Commission [45]. There were 71 general hospitals in Shenzhen in July 2021. The distribution of general hospitals is shown in Figure 3.

**Figure 4:**
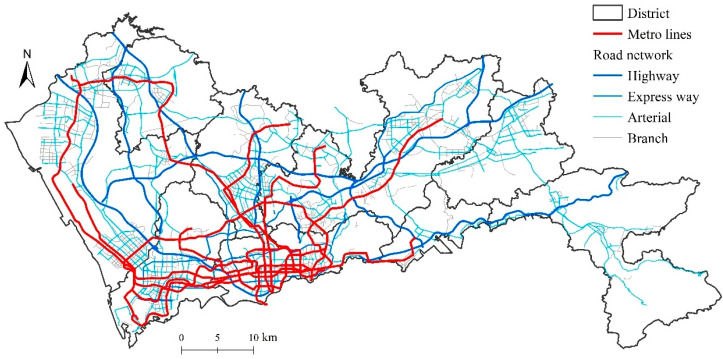
Metro and road networks in Shenzhen.

Figure 4 shows the metro and road networks in Shenzhen. The road network is relatively balanced across ten districts in Shenzhen. Highways and expressways connect all districts, and arterial ways cover most of the developed areas. In contrast, the metro network is more unbalanced. Metro lines are much denser in the central city area and sparser in the peripheral areas. The Pingshan and Dapeng districts are left unconnected to the metro network. This difference suggests that the unbalanced metro network may contribute to a larger disparity in healthcare accessibility. The following analyses will provide further evidence.

## 3. Results

This section first presents the spatial distribution of healthcare accessibility in the five scenarios with various distance measures. It then demonstrates the proportions and spatial patterns of facility- and transport-driven accessibility inequality.

### 3.1. Spatial Distribution of Healthcare Accessibility by Various Distance Measures

**Figure 5:**
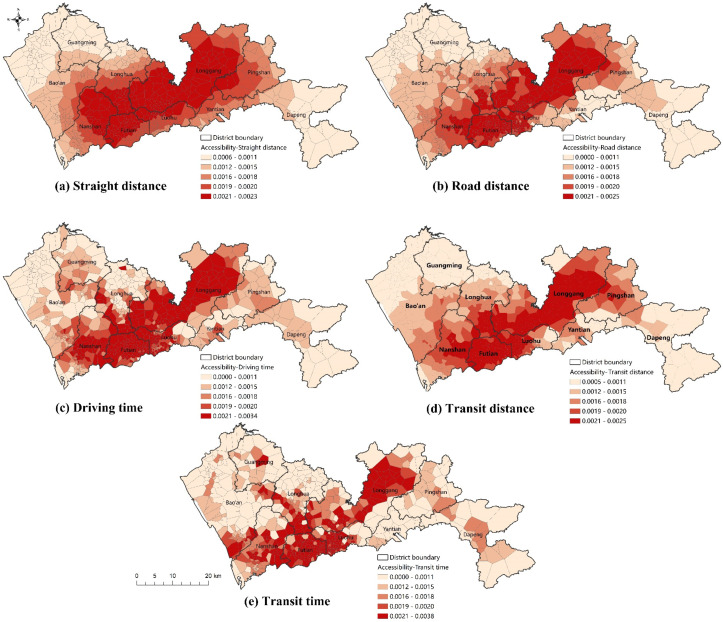
The distribution of healthcare accessibility by various distance measures.

Following the framework in Figure 1, healthcare accessibility was calculated by the Gaussian 2SFCA method for each of the five scenarios. The only difference between these scenarios is the distance measure. The distributions of accessibility in the five scenarios are shown in Figure 5.

In scenario A, where straight distance is used, healthcare accessibility is determined by the distribution of healthcare facilities. From Figure 5a, it can be observed that high healthcare accessibility is concentrated in a corridor area from Nanshan to Longgang. In this corridor area, although a densely distributed population induces a high demand for healthcare, the supply provided by hospitals is also relatively abundant. In contrast, healthcare accessibility remains relatively low in the western and eastern parts of Shenzhen, including Bao’an, Guangming, northern Longhua, and Dapeng.

In scenario B, where distance is measured by road distance, both facility distribution and road network contribute to healthcare accessibility. As shown in Figure 5b, the Nanshan–Longgang corridor area persists in scenario B, but its width is much narrower. This finding indicates that when considering the road network, the spatial disparity in healthcare accessibility becomes more obvious. Both facility distribution and road networks increase the spatial disparity in healthcare accessibility.

In scenario C, travel time by driving is adopted as the distance measure. Compared to scenario B, the actual driving speed differences, which may be caused by road grades and capacities, driving rules, and traffic congestion, are further involved in the measurement of healthcare accessibility. As shown in Figure 5c, the distribution of healthcare accessibility becomes more unbalanced.

Scenario D adopts transit network distance. The distribution of healthcare accessibility by transit distance (Figure 5d) is similar to that by road distance, indicating that the transit network plays a similar role in healthcare accessibility to the road network in terms of network distance.

In terms of travel time, however, a significant difference exists between healthcare accessibility by transit and by driving. As shown in Figure 5c,e, the distribution of healthcare accessibility by transit travel time is obviously more unbalanced than that by driving time. The high-accessibility corridor area in scenario E is the narrowest among the five scenarios. In Figure 5e, it can also be observed that healthcare accessibility is relatively high in the communities along metro lines. This finding demonstrates the superiority of metro transit in facilitating healthcare accessibility.

### 3.2. Proportions of Facility- and Transport-Driven Accessibility Inequality

To quantify the spatial inequality in healthcare accessibility, CV and GC metrics were calculated for each scenario. The results are shown in Table 1. There was a very high correlation coefficient (0.99) between CV and GC. Considering that GC is widely used in measuring equality, the following analyses will be based on GC. In the straight-distance scenario, the GC of healthcare accessibility is 0.130, which is caused by facility distribution. Compared to the straight-distance scenario, the road-distance and transit-distance scenarios further consider the transport network and yield slightly larger GCs (0.147 and 0.156). This finding suggests that transport networks and facility distribution jointly exacerbate the inequality of healthcare accessibility. In the driving-time and transit-time scenarios, GC is much larger (0.212 and 0.256, respectively; 1.63 and 1.97 times that in the straight-distance scenario).

### 3.3. Spatial Patterns of Facility- and Transport-Driven Accessibility Inequality

The distributions of facility- and transport-driven accessibility inequality for each mode can be visualized by comparing the accessibility in various scenarios. First, facility-driven accessibility inequality can be represented by the distribution of straight distance-based healthcare accessibility (Figure 5a). As demonstrated above, facility-related factors tend to facilitate healthcare accessibility along the Nanshan–Longgang corridor area, but lead to low accessibility in the western and eastern parts of Shenzhen.

**Figure 6:**
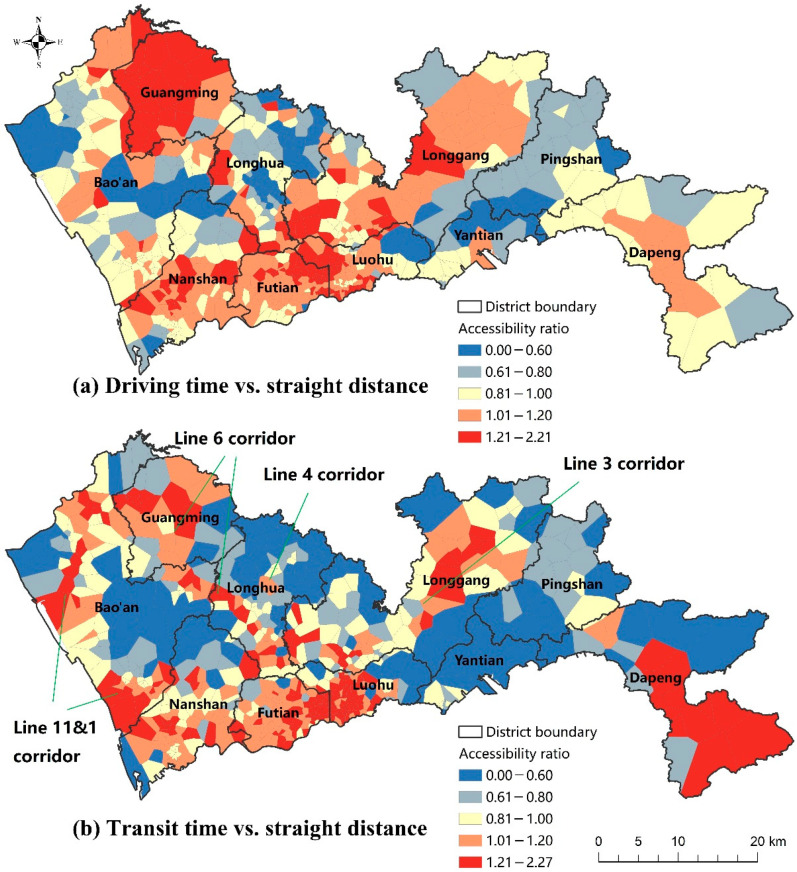
The distribution of the ratios of accessibility by various distance measures.

Second, the contribution of transport-driven accessibility disparity to the driving mode can be measured by the ratio of driving time-based accessibility (scenario C) to straight distance-based accessibility (scenario A). If the ratio is larger than 1, it means that transport-related factors tend to facilitate healthcare accessibility. As shown in Figure 6a, the distribution of large accessibility ratios generally overlaps with the high-accessibility areas, especially in the Nanshan–Longgang corridor area. As a result, inequality in healthcare accessibility is exacerbated by transport inequality for the driving mode. This finding is in accordance with the comparison between GCs and CVs in scenarios A and C in Table 1.

Third, the contribution of transport-driven accessibility inequality to the transit mode can be measured by the ratio of transit time-based accessibility (scenario E) to straight distance-based accessibility (scenario A). As shown in Figure 6b, large accessibility ratios appear in two types of areas. The first type is the central city (Futian, Luohu, and Nanshan districts), where the public transit network (especially metro) is the densest. The second type is the corridor areas along the major metro lines, e.g., the Line 11&1 corridor in Bao’an, the Line 6 corridor in Longhua, Bao’an, and Guangming, the Line 4 corridor in Longhua, and the Line 3 corridor in Longgang. This finding suggests that the public transit network, especially the metro network, significantly reshapes the pattern of healthcare accessibility.

## 4. Discussion

Equal access to healthcare and other services is a recurrent topic in geography and urban planning studies. This study emphasizes the nature of accessibility as a comprehensive concept. In this way, spatial accessibility and its (in)equality depend on not only facility distribution, but also multimodal transport networks. As the results confirm, transport-related factors (in terms of network distance and travel time) contribute to almost half of the spatial inequality in healthcare accessibility.

Although the distribution of healthcare facilities is the same, residents that rely on public transit may face larger inequality in healthcare accessibility than those owning private cars. As an efficient and express transit mode, metro transit significantly facilitates healthcare accessibility in the corridor areas along metro lines. However, the spatial configuration of metro transit is unbalanced due to its huge construction and operation costs. Therefore, it is critical to promote the integration of public transit (especially metro transit) and healthcare facilities.

Promoting spatial equality in accessibility to healthcare and other essential service facilities is the focus of public policy. This study provides a new approach to this goal, which emphasizes that healthcare accessibility is more than a healthcare facility-related issue, as transport-related factors also substantially contribute to accessibility and spatial equality. Therefore, to improve healthcare accessibility and spatial equality, both facility- and transport-related countermeasures are needed.

Overall, the contributions of this study are three-fold. First, it sheds new light on the understanding of the spatial inequality in healthcare accessibility. Second, it provides transferable methods for quantitively decomposing and mapping facility- and transport-driven healthcare accessibility inequality. The methods can also be applied to other types of public service facilities. Third, the findings provide evidence for improvement in spatial equality in healthcare accessibility and the achievement of health equality.

This study still has some limitations. First, this study is focused on the spatial dimension of accessibility and equality, i.e., the distribution of facilities, population, and transport networks and their spatial relationships. It should be kept in mind that accessibility also depends on nonspatial factors, such as economic affordability, institutional barriers, and personal attributes. Second, the heterogeneity of the population (e.g., age, income, and disability) and facility (e.g., size and hierarchy) is not considered in this study. Subpopulations with different socioeconomic characteristics may have heterogeneous preferences for facilities and transport modes. The disparity and inequality in healthcare accessibility among various subgroups should also be investigated in future studies. Third, the findings are based on the case of Shenzhen. It is noteworthy that the components of accessibility and their relative importance depend on the local context of a given city. The applicability of our findings in other contexts and for other facilities needs to be validated in future studies. Fourth, healthcare accessibility is measured based on home locations in this study, while the possibility that people may access healthcare services from other locations (e.g., workplace) is not considered. Fifth, although the online map APIs provide relatively precise estimation of travel time, there are still some factors excluded from the estimation, such as the availability of parking facilities at destinations. Besides, the monetary cost, comfort, and satisfaction of travels are also not considered in this study.

## 5. Conclusions

Spatial equality in accessibility to healthcare and other essential public services is a highlighted policy goal across the world. Researchers and policy makers have made great efforts to achieve this goal. Numerous studies have revealed obvious spatial inequality in accessibility to healthcare and other facilities across various contexts. However, it remains unclear how facility distribution and transport networks contribute to such accessibility inequality.

This study develops an explicit framework for decomposing the spatial inequality in healthcare accessibility into facility- and transport-driven inequality. We set up various scenarios of healthcare accessibility by different distance measures, including straight distance, road distance, driving time, transit distance, and transit time. These scenarios reflect different combinations of facility- and transport-related factors of healthcare accessibility. By comparing the distribution and inequality of healthcare accessibility in these scenarios, the magnitudes and distribution of facility- and transport-driven healthcare inequality can then be estimated.

Using a case study in Shenzhen, China, this study reveals that facility distribution and transport both substantially contribute to spatial inequality in healthcare accessibility. The contributions also differ by various transport modes. For the driving mode, facility-, road network-, and driving time-driven accessibility inequalities account for 61.3%, 8.0% and 30.7% of the overall inequality in healthcare accessibility, respectively. For transit mode, facility-, transit network-, and transit time-driven accessibility inequalities account for 50.8%, 10.1%, and 39.1% of the overall inequality, respectively.

Our analyses also reveal the spatial patterns of facility-, transport network-, and travel time-driven accessibility inequality for each mode. Notably, for the areas that are advantageous in facility-related factors, transport-related factors further strengthen their advantages. In other words, facility distribution and transport networks jointly exacerbate the spatial inequality in healthcare accessibility.

## Figures and Tables

**Table 1:** Spatial inequality in health care accessibility by various distance measures.

Distance Measure	Straight Distance	Road Distance	Driving Time	Transit Distance	Transit Time
CV	0.242	0.269	0.372	0.282	0.449
GC	0.130	0.147	0.212	0.156	0.256

## Data Availability

The healthcare facility data can be obtained from the website of Shenzhen Municipal Health Commission (http://wjw.sz.gov.cn/bmfw/wycx/fwyl/yycx/index.html accessed on 26 April 2022). The travel time data were estimated using the public web API of Baidu Map (http://lbsyun.baidu.com/index.php?title=jspopular/guide/routeplan accessed on 26 April 2022).
